# Ostéite costale tuberculeuse

**Published:** 2010-08-11

**Authors:** Lakranbi Marouane, Smahi Mohamed, Ouadnouni Yassine, Msougar Yassine, Caidi Mohammed, Bouchikh Mohammed, Achir Abdellah, Herrak Laila, El masslout Abderrahmane, Benosman Abdellatif

**Affiliations:** 1 Service de chirurgie thoracique, CHU Hassan II Fes, Maroc; 2 Service de chirurgie thoracique, CHU Rabat, Maroc

**Keywords:** Tuberculose, Ostéite, costale, paroi thoracique, chirurgie thoracique

## Abstract

Nous rapportons un cas de tuberculose costale chez une patiente de 44ans ayant des antécédents de miliaire tuberculeuse. L’atteinte costale était pseudotumorale ayant conduit à une biopsie exérèse chirurgicale de la masse costale dont l’étude histologique est revenue en faveur d’une tuberculose.

Cette observation ainsi que celles de la littérature, confirment les difficultés diagnostiques de cette forme rare de tuberculose.

## Introduction

La tuberculose costale est une forme très rare de tuberculose ostéo-articulaire et revêt parfois des aspects radio-cliniques trompeurs [1]. A travers une observation et une revue de littérature, nous mettrons le point sur les difficultés diagnostiques de cette affection ainsi que sur le rôle de la chirurgie dans la prise en charge de cette pathologie.

## Patient et observation

Il s’agissait d’une patiente de 44 ans, ayant comme antécédents une notion de miliaire tuberculeuse et de méningite tuberculeuse traitées il y a 2ans et qui présentait depuis 1an des douleurs thoraciques droites, sans signes respiratoires et évoluant dans un contexte d’altération de l’état général. L’examen clinique était sans particularités.

La radiographie thoracique avait montré une ostéolyse avec réaction périostée en regard de l’arc postérieur de la 6ème cote droite ( 1).

La TDM thoracique a objectivé une masse hyperdense au dépend de l’arc postérieur de la 6^ème^ cote se développant surtout en endothoracique et ayant un centre hypodense; par ailleurs, il n’existait pas de lésion parenchymateuse ni pleurale ( 2). La scintigraphie osseuse était sans particularités en dehors de l’atteinte costale.

Une biopsie exérèse de l’arc postérieur de la cote pathologique a été effectuée par un abord sous scapulaire permettant de découvrir une tuméfaction triangulaire à la jonction de l’arc moyen et postérieur de la 6^ème^ cote droite à développement surtout endothoracique avec issu de pus.

L’examen anatomopathologique de la pièce opératoire a été en faveur d’une tuberculose caséo-folliculaire.

Les suites opératoires ont été simples et la patiente a été mise sous antibacillaires selon le protocole 2 RHZ/4RH avec bonne évolution.

**1:**
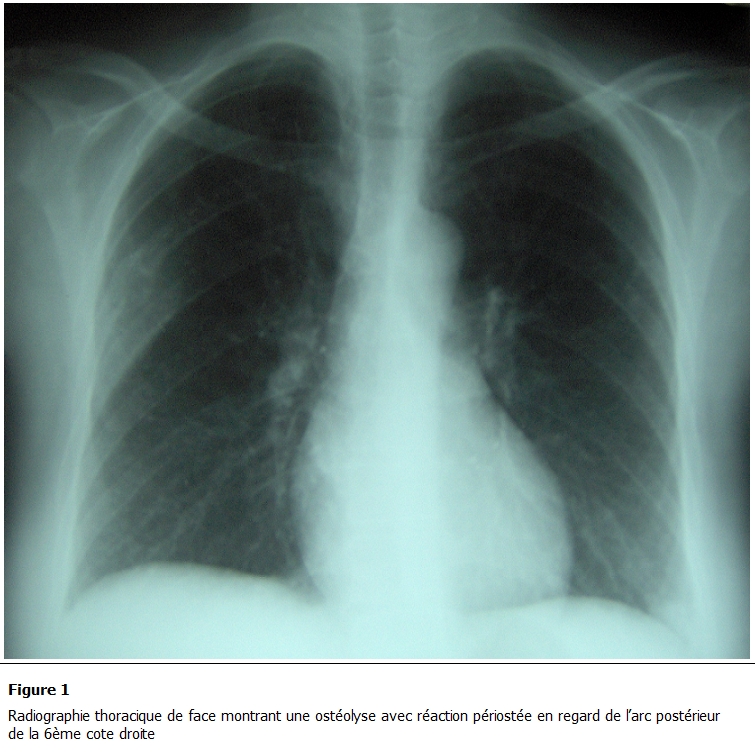
radiographie thoracique de face montrant une ostéolyse avec réaction périostée en regard de l’arc postérieur de la 6^ème^ cote droite.

**2:**
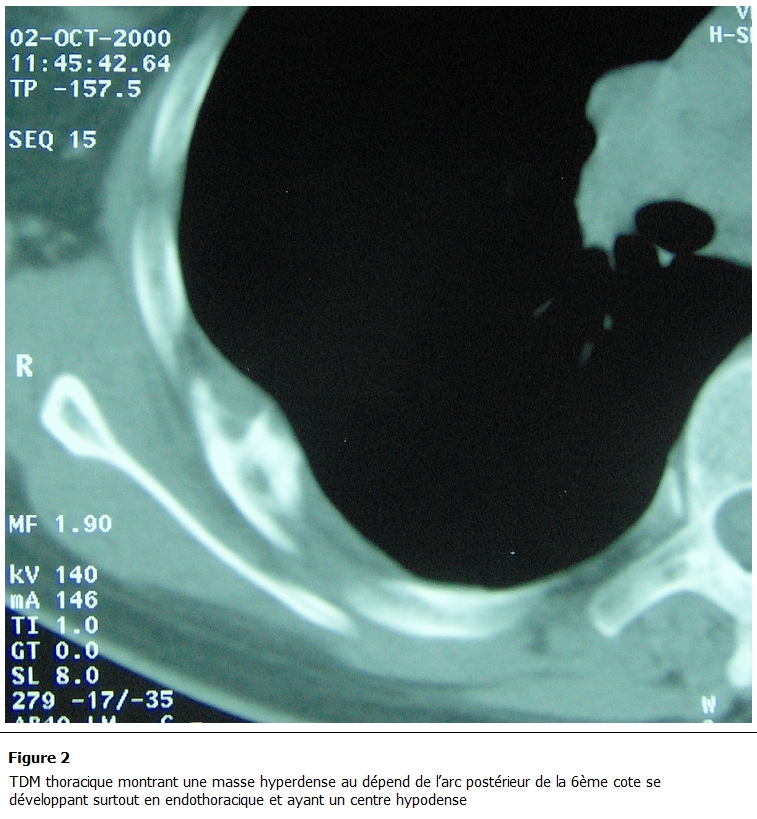
TDM thoracique montrant une masse hyperdense au dépend de l’arc postérieur de la 6^ème^ cote se développant surtout en endothoracique et ayant un centre hypodense.

## Discussion

La tuberculose costale est une forme très rare de tuberculose ostèoarticulaire, représentant 0 à 5% de ses localisations [2]. L’adulte jeune, est le plus souvent atteint avec une légère prédominance masculine [3].

Il s’agit d’une forme paucibacillaire de tuberculose caractérisée par la présence de bacilles de Koch (BK) à croissance lente, en effet les bacilles tuberculeux touchant l’os, peuvent rester dans un état dit dormant durant plusieurs années au sein des lésions caséeuses, échappant ainsi à l’action des antituberculeux ce qui fait que l’atteinte osseuse est le résultat d’une réactivation locale de ces BK dormants [4]; par ailleurs l’atteinte costale est souvent liée à une dissémination hématogène de bacilles tuberculeux à partir d’un foyer viscéral primitif, le plus souvent pulmonaire [3]. Ce fut le cas chez notre patiente.

L’affection peut être soit asymptomatique ou se traduire par des douleurs pariétales, associées le plus souvent à une masse palpable, plus ou moins fluctuante, sans signes inflammatoires, en regard de la côte atteinte. L’atteinte costale peut être isolée ou entrer dans le cadre d’une tuberculose multifocale [5]. Les radiographies standards peuvent être normales au début. Ailleurs, elles peuvent montrer des images à prédominance ostéolytique, parfois associées à des zones d’ostéo-condensation; des fractures pathologiques peuvent être révélatrices; une opacité para osseuse peut être mise en évidence traduisant la présence d’une masse des parties molles [6].

L’échographie peut mettre en évidence une collection des parties molles para-osseuses, ce qui permet de diriger une éventuelle ponction à visée diagnostique et évacuatrice [1].

A la tomodensitométrie (TDM), l’ostéolyse se traduit par des plages de destruction osseuse, à contours nets d’allure parfois pseudo-tumorale associée ou non à une réaction périostée et à un séquestre osseux [7-10].

La scintigraphie permet de rechercher d’autres localisations; cependant, d’une part, elle ne fait pas la part entre une atteinte infectieuse et une origine tumorale et, d’autre part, 35 % des lésions évolutives ne fixent pas le traceur radioactif du fait de leur caractère avasculaire ou purement ostéolytique [2].

Le diagnostic de certitude repose sur les prélèvements bactériologiques au niveau d’un abcès ou d’une fistule, ou sur l’examen histopathologique suite à une biopsie osseuse percutanée. Devant une lésion osseuse non étiquetée, l’abord chirurgical peut s’avérer nécessaire d’autant plus que le diagnostic différentiel inclut les processus tumoraux bénins (granulome éosinophile), malins primitifs (sarcome d’Ewing, ostéosarcome), secondaires, myélomateux, ou encore infectieux non spécifiques [1].

Le traitement repose sur une polychimiothérapie antituberculeuse quadruple de longue durée, alors que l’adjonction d’une résection chirurgicale est limitée aux cas d’échec du traitement médical [4]; inversement d’autres auteurs proposent une résection chirurgicale dès la suspicion du diagnostic dans le but d’avoir une confirmation anatomopathologique mais aussi dans un but thérapeutique d’autant plus que la résection supprime toute néovascularisation assurant ainsi une meilleure distribution des antibaccillaires au niveau costale source d’une meilleure efficacité [11].

## Conclusion

En l’absence d’autres lésions pulmonaires ou extrapulmonaires évocatrices de tuberculose, la symptomatologie radio-clinique oriente surtout vers une lésion néoplasique. De ce fait le recours à une exérèse chirurgicale permet d’obtenir une confirmation histophatologique et aidera à assurer une meilleure efficacité des antibaccilaires.

## Conflit d’intérêts

Les auteurs ne déclarent aucun conflit d’intérêts
